# Insights into the *Morphostenophanes* (Coleoptera, Tenebrionidae) fauna of the Ailao Mountains: A new species, key, and distributional pattern of the sympatric species

**DOI:** 10.3897/zookeys.1282.184301

**Published:** 2026-06-15

**Authors:** Zi-Chun Xiong, Zheng-Quan Ye, Zhi-Yun Lu, De-Yao Zhou

**Affiliations:** 1 Ailaoshan Subtropical Forest Ecosystem Observation and Research Station of Yunnan Province, Xishuangbanna Tropical Botanical Garden, Chinese Academy of Sciences, Jingdong, Yunnan 676209, China Ailaoshan Subtropical Forest Ecosystem Observation and Research Station of Yunnan Province, Xishuangbanna Tropical Botanical Garden, Chinese Academy of Sciences Jingdong China; 2 Chuxiong Management Bureau of Ailaoshan National Nature Reserve, Chuxiong 675000, China Chuxiong Management Bureau of Ailaoshan National Nature Reserve Chuxiong China; 3 Dacheng Xiaochong Studio, Songjiang District, Shanghai 201601, China Dacheng Xiaochong Studio Shanghai China

**Keywords:** China, darkling beetle, identification key, new taxon

## Abstract

*Morphostenophanes ailaoshanensis***sp. nov**. is described, which is the fourth species distributed in the Ailao Mountains in Yunnan, China. Detailed illustrations of the habitus and diagnostic characters of the new species are provided. Furthermore, an identification key to all four *Morphostenophanes* species recorded in the Ailao Mountains is presented, supplemented by geographical and altitudinal distribution maps.

## Introduction

The darkling beetle genus *Morphostenophanes* Pic, 1925 (Tenebrionidae, Stenochiinae) currently comprises 33 species-level taxa across six species groups, following the comprehensive revision by Zhou (2020) and recent taxonomic additions from Xizang, China (Zhou et al. 2025) and northern Vietnam (Huang 2025). Characterized by their flightless nature and restricted dispersal capabilities, these beetles exhibit high levels of localized endemism, with Yunnan Province, China, serving as the primary centre of their diversification.

The Ailao Mountains (Ailaoshan National Nature Reserve) in central Yunnan constitute a significant biodiversity hotspot for *Morphostenophanes*. Although three species were previously documented from this range, recent surveys have yielded a fourth congeneric taxon, further emphasizing the region’s remarkable localized diversity. In the present paper, we describe *Morphostenophanes ailaoshanensis* sp. nov. and provide a diagnostic key to the four species now recorded from the Ailao Mountains. Furthermore, based on newly collected material, we characterize their spatial and altitudinal distribution using updated maps and elevational profiles to clarify the ecological patterns of these endemics within this montane system.

## Material and methods

The holotype and paratypes designated in this study are labelled with red and yellow labels respectively. Specimens examined in the current study are deposited in the following institutions, museums or private collections. Abbreviations for collections are:

**XTBG** Xishuangbanna Tropical Botanical Garden, Chinese Academy of Sciences, Mengla, Yunnan, China;

**SNUC** Insect Collection of the Shanghai Normal University, Shanghai, China.

Habitus photographs were taken using a Nikon^®^ Z7 digital camera with a Laowa^®^ Laowa 100 mm f/2.8 2× macro lens and stacked using Zerene Stacker v. 1.04. Smaller characters were photographed using the same camera equipped with Laowa^®^ 25 mm f/2.8 2.5–5× ultra macro lens. All photographs were refined in Adobe Photoshop^®^ CC2019.

The following abbreviations are applied in measurements: **EL**—length of elytra along midline; **EW**—maximum width of elytra; **OI**—ocular index (Campbell and Marshall 1964); **PL**—length of pronotum along midline; **PW**—maximum width of pronotum. Body length was measured from the middle of the anterior margin of clypeus when the head is in a natural position to the apex of elytra, and body width equals to EW.

## Taxonomy

### Family Tenebrionidae Latreille, 1802

**Subfamily Stenochiinae W. Kirby, 1837**

**Tribe Cnodalonini Oken, 1843**

**Genus *Morphostenophanes* Pic, 1925**

#### Morphostenophanes ailaoshanensis

Taxon classificationAnimaliaColeopteraTenebrionidae

Xiong & Zhou sp. nov.

1EBF6926-5D8D-5C34-ABC4-035A7621F49F

https://zoobank.org/16E4B189-FB3A-4E01-9927-D3D198AED78E

Figs 1, 2, 3

##### Chinese common name.

哀牢山窄亮轴甲.

##### Type material.

***Holotype*: China**: • ♂, ‘China: Yunnan, Chuxiong Yi Pref., Ailashan N.R., Shuangbai County, Heizhushan (24.3425°N, 101.2874°E), alt. 2748 m., 2025.iii.6, Zi-Chun Xiong leg. 哀牢山保护区黑竹山瞭望台熊紫春采’ (XTBG). ***Paratypes*: China**: • 1 ♀, same data as for holotype (XTBG), • 2 ♂♂, 1♀, ditto (SNUC), • 1♀, ‘China: Yunnan, Yuxi City, Xinping County, Mount Daxueguoshan, 24.167697°N, 101.371103°E, alt. 2840 m, 2026.iv.21, Zi-Chun Xiong leg. 云南玉溪市新平县大雪锅山熊紫春采’ (XTBG).

##### Diagnosis.

A small species. Dorsum and venter dark metallic green; elytra with a strong cupreous lustre and distinct ring-like depressions bearing purplish-violet reflections. Antennomere III at least 1.1 times as long as IV. Elytra strongly convex in both sexes.

##### Description.

**Figure 1.**
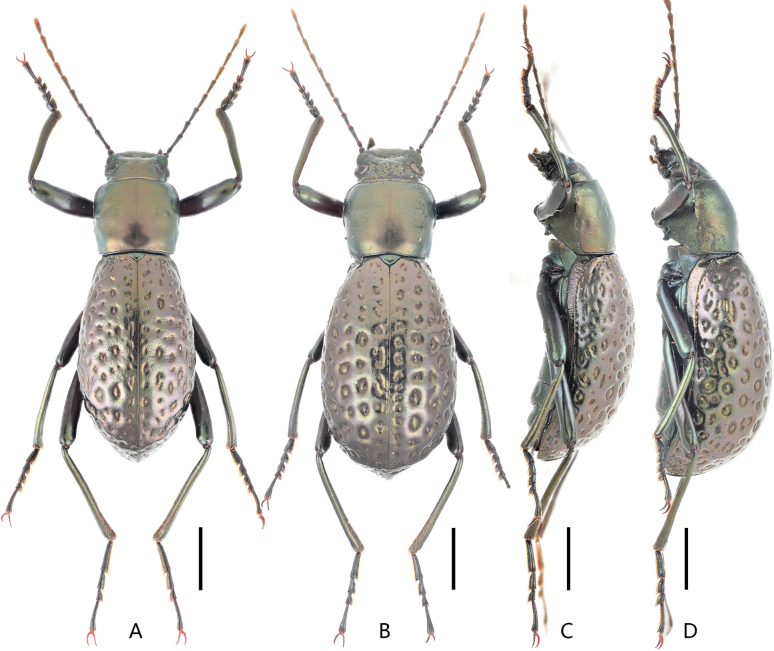
Habitus of *Morphostenophanes ailaoshanensis* sp. nov. **A, C**. Male, holotype; **B, D**. Female, paratype. In dorsal (**A, B**) and lateral (**C, D**) view. Scale bars: 3.0 mm.

**Holotype male**. Habitus (Fig. 1A, C) slender, length 15.7 mm, width 5.6 mm, strongly convex, noticeably constricted between pronotum and elytra. Body dark greenish, with metallic lustre, elytra cupreous with purplish-violet ring-like depressions; antennomeres 7–11 bearing brown pubescence.

**Figure 2.**
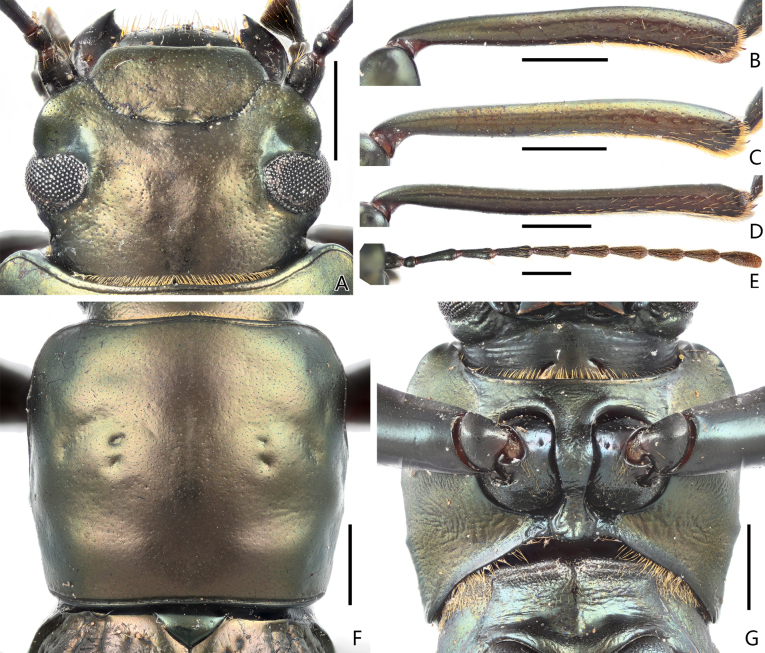
External characters of *Morphostenophanes ailaoshanensis* sp. nov., in male. **A**. Head in dorsal view; **B**. Protibia in dorsal view; **C**. Mesotibia in ventral view; **D**. Metatibia in dorsal view; **E**. Antenna in dorsal view; **F**. Prothoraces in dorsal view; **G**. Same, in ventral view. Scale bars: 1.0 mm.

Head (Fig. 2A) transversely subquadrate, sparsely and coarsely punctate; clypeus transversely hexagonal, gently bent downwards in front, with anterior margin nearly straight, clypeal transverse impression short; frontoclypeal suture depressed, widely U-shaped; genae strongly raised, depressed before eyes, strongly and roundly produced anterolaterally; frons broad, with anterior part gradually sloping forwards; eyes transversely reniform, strongly convex laterally; inner ocular sulci finely depressed; tempora weakly convex, finely wrinkled. OI = 69.0. Antennae (Fig. 2E) slender, exceeding basal third of elytra, with antennomeres weakly thickened to each apex; antennomere III 1.1 times as long as IV; relative lengths of antennomeres from base to apex: 1.00: 0.54: 1.43: 1.29: 1.40: 1.40: 1.40: 1.37: 1.29: 1.29: 1.51. Mentum subtrapezoidal, finely and sparsely punctate.

Pronotum (Fig. 2F) quadrate, widest slightly before the middle; anterior margin nearly straight, with markedly clear marginal sulcus; PW/PL = 1.17; lateral margins curved, with lateral sulci thin, visible along anterior half in dorsal view; posterior margin feebly sinuate, with markedly clear marginal sulcus; anterior angles rounded, posterior angles obtusely rounded; disc convex in middle, shagreened, finely and sparsely punctate, with two distinct, rounded impressions laterally. Scutellum triangular, glossy.

Elytra fusiform, widest slightly behind middle, strongly convex, highest near anterior 1/3, and bearing five rows of ring-like depressions, which are elevated at each middle, and rows are interrupted in part; EL/EW = 1.79; surface sparsely, finely punctate.

Prosternum shagreened, finely and sparsely punctate; prosternal process declivous, pointed at apex; hypomeron weakly rugulose, shagreened. Metaventrite glossy, metaventral anterior process weakly wrinkled. Abdomen depressed, surface somewhat rough, densely and finely punctate, with ventrites III and IV sulcate at sides; ventrite V rimmed, rim interrupted in middle.

Legs slender. Protibiae (Fig. 2B) curved at apical 2/5, pubescent in apical half of inner margin; mesotibiae (Fig. 2C) weakly curved at apical third, pubescent in apical half of inner margin; metatibiae (Fig. 2D) weakly sinuous, pubescent in apical 3/5 of inner margin.

**Figure 3.**
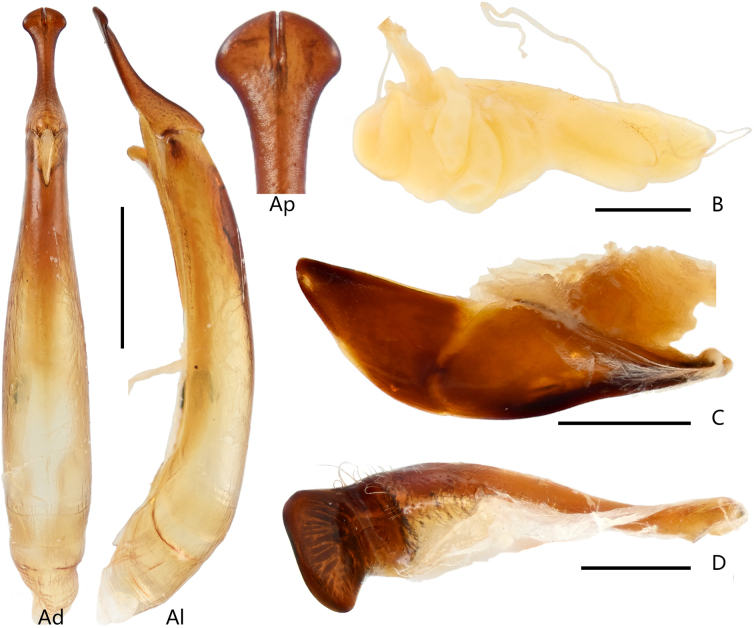
Anatomic characters of *Morphostenophanes ailaoshanensis* sp. nov. **A**. Aedeagus in dorsal (**d**) and lateral view (**l**), and enlarged parameres apices (**p**); **B**. Bursa copulatrix in female; **C**. Ovipositor in female; **D**. Male sternite 8 in lateral view. Scale bars: 1.0 mm (**A–C**); 0.5 mm (**D**).

Aedeagus (Fig. 3A) elongate, curved in lateral view; parameres slender, straightly producing in lateral view, 0.24 times as long as the total length, with apices flabellated. Sternite VIII (Fig. 3D) with apical lobes rectangular in lateral view, with interior margins rounded, cornered at lowest point.

**Female**. Habitus (Fig. 1B, D) stouter than the male, BL = 18.8–16.5 mm. Antennomere III 1.2 times as long as IV, PW/PL = 1.18–1.22; elytra more convex, EL/EW = 1.55–1.65, highest at middle; abdomen straight in lateral view. Ovipositor (Fig. 3C) knife shaped, gradually narrowing apically, fused coxites shorter than baculus.

##### Variability.

The male paratypes with BL = 14.5–16.1 mm; OI = 57.5–59.0. Pronotum with PW/PL = 1.15. Elytra with the ratio of EL/EW = 1.85.

##### Comparative notes.

Such small-sized species of *Morphostenophanes* were previously only known from the jendeki- and *metallicus*-groups. The new species is clearly more similar to *M. minor* Zhou, 2020, which is currently assigned to the *jendeki*-group. It can be distinguished from the latter by the following characters: body primarily metallic green (metallic cupreous in *M. minor*); elytral ring-like depressions significantly deeper; antennomere III at least 1.1 times as long as IV (these two antennomeres are subequal in length in the latter); the apices of parameres without apical marginal carina; and the distinct shape of the apical lobes of sternite VIII.

##### Distribution.

China: Yunnan.

##### Etymology.

The new species is named after its type locality, Ailaoshan National Nature Reserve.

###### Other species recorded from Ailao Mountains

#### Morphostenophanes sinicus

Taxon classificationAnimaliaColeopteraTenebrionidae

Zhou, 2020

5D1C1DCB-2AD7-5D09-8DB8-70D2E7A8962C

##### Chinese common name.

中华窄亮轴甲.

##### Material examined.

• 1 ♂, ‘China: Yunnan, Chuxiong Yi Pref., Ailashan N.R., 24.53334243°N, 101.0327751°E alt. 2648 m, 2023.viii.30, Zi-Chun Xiong leg.’ (XTBG), • 1♂, 1♀, ditto except ‘24.56011077°N, 101.00189987°E, alt. 2354 m, 2024.xi.14.’ (XTBG), • 14 ♂♂, 2 ♀♀, ditto except ‘24.85923711°N, 100.80533333°E, alt. 2480 m, 2025.iii.30.’ (XTBG).

#### Morphostenophanes yunnanus

Taxon classificationAnimaliaColeopteraTenebrionidae

Zhou, 2020

A09B08B6-38BE-5419-A67D-90FC76D9DB3F

##### Chinese common name.

滇中窄亮轴甲.

##### Material examined.

• 6 ♂♂, 4 ♀♀, ‘China: Yunnan, Chuxiong Yi Pref., Ailashan N.R., 24.56011077°N, 101.00189987°E, alt. 2354 m, 2024.xi.14, Zi-Chun Xiong leg.’ (XTBG), • 11 ♂♂, 6 ♀♀, ditto except ‘24.85923711°N, 100.80533333°E, alt. 2480 m, 2025.iii.30’ (XTBG).

#### Morphostenophanes chongli chongli

Taxon classificationAnimaliaColeopteraTenebrionidae

Zhou, 2020

E764C060-041E-55D9-A27E-7B9BBEDF6382

##### Chinese common name.

火神窄亮轴甲指名亚种.

##### Material examined.

• 1 ♂, 1 ♀ ‘China: Yunnan, Chuxiong Yi Pref., Ailashan N.R., 24.48825245°N, 100.99112351°E, alt. 2029 m, 2024.iv.26, Zi-Chun Xiong leg.’ (XTBG).

### Key to the species of *Morphostenophanes* from the Ailao Mountains

**Table:** 

1	Elytra densely covered with irregular, scattered tubercles	***M. chongli chongli* Zhou, 2020**
–	Elytra densely bearing five rows of ring-like depressions with raised centres	**2**
2	Body size relatively small (length 14.5–18.8 mm); elytra covered with deep, ring-like depressions bearing purplish-violet reflections	***M. ailaoshanensis* sp. nov**.
–	Body size larger (length usually >18 mm); elytra covered with ring-like depressions without purplish-violet reflections	**3**
3	Elytra widest behind middle, bearing small and dense ring-like depressions; female ovipositor shortened	***M. yunnanus* Zhou, 2020**
–	Elytra widest in middle, elytra bearing large, partially confluent ring-like depressions; female ovipositor elongated	***M. sinicus* Zhou, 2020**

**Figure 4.**
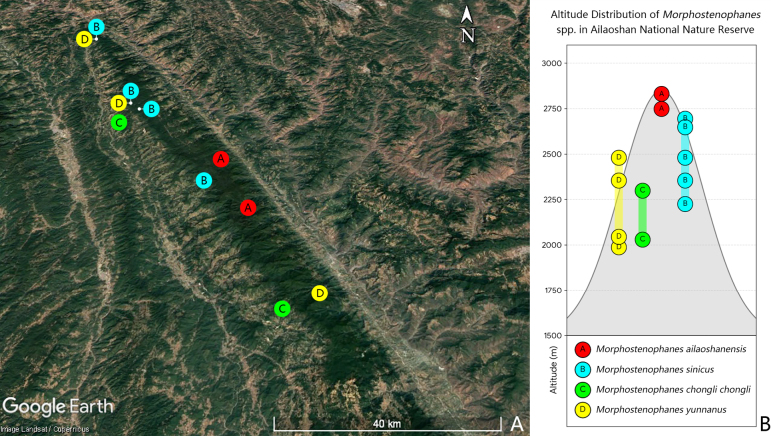
Occurrence of the four *Morphostenophanes* species in the Ailaoshan National Nature Reserve. **A**. Geographic distribution; **B**. Elevational range.

Distributions of all *Morphostenophanes* species recorded from the Ailao Mountains are plotted in Fig. 4.

## Discussion

The discovery of *Morphostenophanes ailaoshanensis* sp. nov. provides a new perspective on the classification and evolutionary history of the *jendeki*-group. Traditionally, the *jendeki*-group was defined primarily by their relatively small body size (usually <17 mm) and their restricted distribution in high-altitude habitats between 2500 m and 3500 m (Zhou 2020). Most members, such as *M. jendeki jendeki*, *M. jendeki similis*, *M. crassus*, and *M. tanikadoi*, exhibit a characteristic elytral morphology featuring either smooth surfaces between irregular punctures or rugose textures. However, the inclusion of taxa with drastically different elytral structures—such as the tuberculated *M. tuberculatus* and the ring-depressed *M. minor*—has already hinted at the morphological heterogeneity within this group (Zhou 2020).

Our morphological analysis reveals that *M. ailaoshanensis* sp. nov. possesses a mosaic of characters that do not fully align with the current definition of the *jendeki*-group. Notably, its aedeagus structure differs from the typical jendeki-type, yet its overall habitus and elytral patterns closely resemble *M. minor*. This combination of characters suggests a transitional state, potentially representing a lineage evolving from an ancestor of the *elegantulus*-species group towards a miniaturized form adapted to high-altitude environments.

This find leads us to hypothesize that the so-called *jendeki*-group may not be a monophyletic lineage, but rather a polyphyletic or paraphyletic assemblage of species that have independently undergone “high-altitude miniaturization”. Such evolutionary trends are well documented in various montane insect taxa, where extreme alpine conditions—characterized by low temperatures, limited oxygen, and shortened foraging windows—favour smaller body sizes to optimize developmental rates and thermoregulation (e.g. in certain Carabidae and Curculionidae; Mani 1968).

In the case of *Morphostenophanes*, the ancestor of the *elegantulus*-group, which typically possesses larger bodies and prominent ring-like depressions at mid-elevations, may have colonized higher altitudes multiple times. This colonization likely triggered a convergent reduction in body size while retaining or modifying ancestral elytral structures, like the ring-like depressions seen in *M. ailaoshanensis* and *M. minor*. Our findings demonstrate that the current *jendeki*-group is an artificial ecological grade rather than a natural monophyletic clade. Consequently, we propose a formal reconstruction of this assemblage, partitioning it into three distinct lineages based on their divergent elytral morphology: the jendeki-species group (*sensu stricto*), the newly established minor-species group, and the reassignment of *M. tuberculatus* to the *chongli*-group.

### *Morphostenophanes jendeki* species group (*sensu stricto*)

The jendeki-species group (*sensu stricto*) is redefined here by the presence of irregular, short, strial punctures on the elytra.

*Morphostenophanes crassus* Zhou, 2020

*Morphostenophanes jendeki* Masumoto, 1998

*Morphostenophanes jendeki similis* Masumoto, 1998

*Morphostenophanes planus* Zhou, 2020

*Morphostenophanes tanikadoi* Masumoto, 1998

### *Morphostenophanes minor* species group

The minor-species group is characterized by their relatively small body, elytra bearing five rows of ring-like depressions, elevated at each middle and partially interrupted.

*Morphostenophanes minor* Zhou, 2020

*Morphostenophanes ailaoshanensis* Xiong & Zhou, sp. nov.

### *Morphostenophanes tuberculatus* Gao & Ren, 2009

*Morphostenophanes tuberculatus* is hereby transferred to the chongli-species group due to its irregular and densely distributed elytral tubercles, a character state shared with *M. chongli* and *M. chongli glaber*.

## Supplementary Material

XML Treatment for Morphostenophanes ailaoshanensis

XML Treatment for Morphostenophanes sinicus

XML Treatment for Morphostenophanes yunnanus

XML Treatment for Morphostenophanes chongli chongli

## Additional information

### Artificial Intelligence (AI) use

Regarding the use of AI in the preparation of this manuscript, the authors declare the following:

During the preparation of this work, the authors used Gemini 3 Pro (Google) exclusively for linguistic refinement, spelling, and formatting checks, and for improving the readability of the English text. The authors affirm that this AI tool was not involved in the generation of scientific data, the interpretation of morphological characters, or the formulation of taxonomic decisions (e.g. diagnoses and descriptions). The authors carefully reviewed all AI-suggested edits and retain full intellectual property rights and responsibility for the scientific content, integrity, and accuracy of the publication.

### Author ORCIDs

Z.-C. Xiong https://orcid.org/0009-0008-5639-9926

Z.-Y. Lu https://orcid.org/0000-0001-9488-3366

D.-Y. Zhou https://orcid.org/0000-0002-2014-9967

### Data availability

All of the data that support the findings of this study are available in the main text.
